# Influence of autistic traits and communication role on eye contact behavior during face-to-face interaction

**DOI:** 10.1038/s41598-024-58701-8

**Published:** 2024-04-08

**Authors:** Max Thorsson, Martyna A. Galazka, Jakob Åsberg Johnels, Nouchine Hadjikhani

**Affiliations:** 1 Gillberg Neuropsychiatry Centre, Institute of Neuroscience and Physiology, University of Gothenburg, Gothenburg, Sweden; 2 Division of Cognition and Communication, Department of Applied Information Technology, University of Gothenburg, Gothenburg, Sweden; 3 Section of Speech and Language Pathology, Institute of Neuroscience and Physiology, University of Gothenburg, Gothenburg, Sweden; 4 Athinoula A. Martinos Center for Biomedical Imaging, Massachusetts General Hospital, Harvard Medical School, Boston, MA USA

**Keywords:** Face-to-face, Deep learning, Eye contact, Gaze convergence, Autism, Human behaviour, Behavioural methods

## Abstract

Eye contact is a central component in face-to-face interactions. It is important in structuring communicative exchanges and offers critical insights into others' interests and intentions. To better understand eye contact in face-to-face interactions, we applied a novel, non-intrusive deep-learning-based dual-camera system and investigated associations between eye contact and autistic traits as well as self-reported eye contact discomfort during a referential communication task, where participants and the experimenter had to guess, in turn, a word known by the other individual. Corroborating previous research, we found that participants’ eye gaze and mutual eye contact were inversely related to autistic traits. In addition, our findings revealed different behaviors depending on the role in the dyad: listening and guessing were associated with increased eye contact compared with describing words. In the listening and guessing condition, only a subgroup who reported eye contact discomfort had a lower amount of eye gaze and eye contact. When describing words, higher autistic traits were associated with reduced eye gaze and eye contact. Our data indicate that eye contact is inversely associated with autistic traits when describing words, and that eye gaze is modulated by the communicative role in a conversation.

## Introduction

Mutual eye gaze is an important nonverbal signal that supports our understanding of others and structures our discourse. From infancy, individuals derive insights into others' interests and intentions through eye contact^1^, and among many species, including humans, direct eye contact may in certain contexts also convey potential threats or social dominance^2^. Within a communicative context specifically, although likely beyond the level of conscious control, eye gaze is used not only to initiate verbal exchange with another person, but also to signal that we are attending to what they are saying and that we are expecting them to speak, effectively using eye contact to structure the conversational flow^3^. The roles we have in the discourse may also affect our gaze patterns. For example, listeners tend to maintain longer gazes at speakers, while speakers offer quicker glances toward their listeners^4–7^. Furthermore, gazing at the listener during question initiation allows the speaker to assess comprehension, beliefs, and intentions, beyond merely confirming attention^8^.

In addition to gaze patterns, gaze avoidance has been examined as another aspect of conversation. One interesting hypothesis suggests that looking away while speaking or listening is a strategy used to reduce mental load when having difficulty encoding^4^. Support for this hypothesis comes from studies examining breaks in eye contact during conversations, particularly when speakers used vocabulary that referred to mental processes^9^.

The idea of eye gaze avoidance due to increased cognitive load is reminiscent of autobiographical reports from autistic individuals who are known to have difficulties in social communication^10^. In one recent study, Trevisan et al.^10^ analyzed comments on various online forums from individuals with self-declared autism, who reported that eye contact is often accompanied by physiological reactions and sensory overload. Indeed, in another study^11^, a thematic analysis from interviews with individuals with autism found that being overloaded during face-to-face interaction is one of the reasons individuals with autism report avoiding eye contact.

Importantly, much like individuals with an autism diagnosis, individuals who score high on autistic traits in the general population^12^ also exhibit challenges in social communication (e.g., Itier and Batty ^13^). A variety of tasks focused on referential communication, in which a person has to convey information so that their partner understands particular referents, have highlighted that individuals with autism or with high autistic traits have similar difficulties with providing relevant information for the listener to guess a referent among competitors^14,15^.

Novel developments in the use of eye tracking during face-to-face interactions, discussed in more detail below, have further specified the nature of these types of interactions. In one study, eye-tracking analysis confirmed previous reports of reduced amount of mutual eye contact in individuals with autism^16^. In their recent study, Ross et al.^17^ found that reduced eye gaze during conversation was inversely associated with increased scores on the short version (AQ-10) of the autism spectrum quotient (AQ). Here, interestingly, the amount of gaze to the eyes of the conversational partner was related to his/her role in the discourse—the subject looked more at their partner when listening than when speaking, again confirming prior research^4–7^. These findings underscore the importance of considering not only individual differences in autistic traits but also contextual factors related to the communicative role when unraveling the complexities of eye gaze in face-to-face communication. Another study investigated the impact of so-called gaze anxiety, characterized by avoidant gaze behaviour^18,19^. That study, however, did not find a relationship between gaze anxiety and behavioral evidence of gaze avoidance in face-to-face interaction^19^. Besides a need for replication of these findings, the relationship between eye contact discomfort and conversational role in the discourse was not specifically explored in that study.

## Quantification of eye gaze in face-to-face interactions

Quantifying eye contact with precision and accuracy is challenging. A recent review outlined various measurement methods that have been applied during the last decades to study eye contact during conversations^20^. For example, it reported that eye contact with a person or observer has been calculated using a coding sheet for a scale-based estimate^21,22^, a timer to estimate total looking time^23^, or an event recorder to determine frequency and duration^24^.

In recent years, advancements in eye-tracking technology have begun to enable researchers to study face-to-face gaze in more detail. Technological solutions such as video cameras or eye trackers placed between or behind participants can provide more exact and objective measures than fully manual labelling^25,26^. Recently, the use of eye-tracking glasses has provided a versatile option for gaze estimation in interactions without extensive equipment^17,27–29^. An important question in this regard is the extent to which results using face-to-face eye-tracking setups reflect findings from previous two-dimensional presentations^30–32^, and conversely, whether experimental settings can be said to reflect face-to-face interactions of daily life^17,27,33^, which is not as straightforwardly defined and may be discussed^34^.

Relatedly, many current solutions also have their limitations. Eye-tracking glasses, for example, are less tolerated by those with sensory and attention issues, and therefore may make the interactions different from interactions in daily life^35–37^. In addition, accuracy and stability to movement have seldom been high enough for measuring where gaze is directed to specific areas of the face, such as the eyes^38,39^. Furthermore, using screens placed behind or between individuals is a more non-invasive form of gaze tracking, but it introduces restrictions and a so-called “eye-contact parallax”^40^, i.e., the impression that when you look another person in the eye, they will see it as if you look away, due to the offset in actual and displayed perspective projection (as can be evident during interactions on Zoom or Skype). Adjustments to this method, such as the use of half-silver mirrors, aim to reduce this offset^16^.

Finally, scene-based approaches such as using commercial eye trackers on tables with cameras from above may be experienced as less intrusive, but these are also not immune to parallax error, which occurs when the participants move from the calibrated plane^25,26,41^.

In sum, while all setups present both benefits and limitations, the general consensus is that eye tracking offers more precise measurements compared to estimation or coding sheet techniques, enabling researchers to objectively quantify eye contact with refined criteria and ultimately facilitating replication.

## The current study

We have designed, made, and developed a dual-camera system that accurately classifies gaze between interlocutors in face-to-face interactions^42^. The setup—called i + i—was developed with neurodevelopmental populations in mind, without additional cameras near the participants or wearables. In the current study, we wanted to test if our experimental setup provides similar findings of differences in eye gaze between describing and listening roles^17,43^. Also, we wanted to see if our novel system could be used to corroborate previous results of eye contact characteristics related to difficulties associated with autism and autistic traits. We finally also specifically explored gaze patterns as a function of participants’ self-reported eye contact discomfort with this novel system, since prior research^19,44,45^ has revealed mixed findings regarding this association.

Specifically, we used the novel setup to investigate how eye gaze and mutual eye contact relate to autistic traits in a cooperative task that involves referential communication. Our research aims are outlined as follows:

1. Understanding eye gaze during face-to-face interaction, including the percentage of eye gaze, in a referential communication turn-taking task involving one person describing words while the other individual is listening and guessing what these words are.
2. Exploring *associations* between self-reported autistic traits, eye contact discomfort and eye gaze, including mutual eye contact, in this scenario.

Based on previous research^4,6,7,17^, we expect more gaze to the experimenter’s eyes when the participants guessed than when they described words as there would be more focus on the experimenter when he provides verbal input. Also, we examine how the “mutuality” of eye gaze manifests itself during this communicative exchange. That is, we uniquely explore how eye gaze data from the two interlocutors overlap in time. Finally, we anticipated a relationship between autistic traits and diminished eye gaze toward the experimenter's eye area, including a reduction in mutual eye contact. Given that discomfort with eye contact is a common issue for individuals with autism and high autistic traits, but not exclusively so, we also examined the distinct impact of reported eye contact discomfort on attention to the eyes of the communication partner.

## Methods

### Subjects

Twenty participants (8 males, 40%), with a mean age of 28.4 years (range 12.8–36.4 years) were recruited from an ad on the University Research Center for Neurodevelopmental Disorders website or word-by-mouth, for the face-to-face assessment with the experimenter (a 28-year-old licensed physiotherapist male, without autism diagnosis and with an AQ score below the cut-off). Two participants were below 18 years old, and therefore their parents filled out the age-appropriate adolescent version of AQ (for ages 12–15 years) ^46^. Participants received two movie tickets (worth ~ 240 SEK/21 EUR) as compensation for participating in the experiment. We expected individuals with varying degrees of autistic traits to show interest in participation. We did not gather data on actual diagnoses but relied on ratings of autistic traits (see details below). The average AQ score for this sample was 19.5 (median: 18.8, range: 12.8–29.0), which was somewhat higher relative to the non-clinical population mean of 15.3 points. Two participants scored ≥ 27.0 points, which is above what has been suggested as a cut-off for autism, with acceptably high sensitivity and specificity^47^. Six of the participants also reported discomfort with eye contact in the interview. Importantly, neither the AQ score nor self-reported eye contact discomfort for each participant was known to the experimenter.

### Apparatus

**Figure 1:**
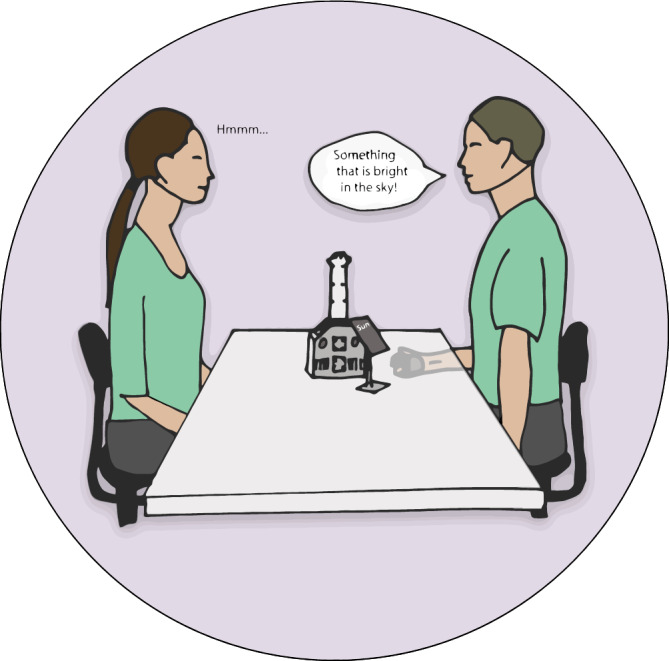
Graphical illustration of the experimental setup and task, see camera system on table and Android phone on the stand displaying the word. The describer’s hand, holding the wireless mouse, is below the table. Cameras, positioned centrally at 29.5 cm above the table, angled at 30°.

We have recently shown that an adaptation of current deep-learning methodologies for eye tracking^48,49^, together with a dual-camera system, can determine gaze to discriminate specific facial areas and quantify movement synchronization^42^. The camera system has been developed with neurodevelopmental disorders in mind; it is minimal, does not include any wearables, such as eye-tracking glasses, and is located on a table between two individuals without covering the face of the person in front of them, see Fig. 1. The camera system recorded videos of the two interlocutors at 60 Hz*.*

### Procedure

Before the experiment, written consent was received from all participants and their caregivers when applicable. Ethical approval for the study had, prior to the data collection, been received from the Swedish Ethical Review Authority. We declare that all methods were performed in accordance with the relevant guidelines and regulations.

Previously mentioned studies^14,15,50^ have used tasks that require the participants to describe a referent (e.g., a face or toy) among competitors (e.g., other faces or toys)—yet these tasks instinctively prompt the listener to look at their options, which potentially can hinder spontaneous eye contact, known to deviate in autism^51^.

Another referential communication task that circumvents this issue simply is one in which one individual describes a word to the other person, who will have to guess what this word is within a given time frame. In the present study, we used words from the Swedish pocket version called “Med Andra Ord”^52^, which is based on a word explanation task in the board game called “Alias”^53^. For example, if you get a card with the word “sun”, you could describe it to the other person as “something bright in the sky”, to help the other person guess the right word. If the other person says “star” you could then say, “almost, but it is only visible during the day".

In our study, each dyad consisted of the experimenter and one participant sitting on a chair, unconstrained, approximately 60–80 cm apart (which was the optimal distance for the camera system to capture the participants' faces). Based on the estimated 3D head locations (determined by facial landmarks), the participants and experimenter were seated approximately 65 cm apart on average. Participants were asked to sit as close as possible to the table, however without it being experienced as uncomfortable. The experimenter adapted his posture to ensure that the participants' knees were not in contact with each other. The heights of the chairs were adjusted so that their eyes were at the same level, ~ 60 cm above the table. The experimenter was one of the two interlocutors, in order to provide a more comparable and predictable interaction across participants.

An Android app was made to provide a temporal structure for the experiment and to minimize the effect of the individual's looks at the word card. A word that was chosen randomly from 588 words, and was displayed for a duration of 0.5 s, to encourage mutual gaze. Used words were put aside so that all the words were new for the experimenter and participant. The experimenter and the participants alternatively played the role of the describer, starting with the experimenter. Between task switches, participants were allowed to ask questions or pause if they wanted. This was included in the protocol to ensure that the participants understood the task and that they could pause if they were uncomfortable. If the word was accurately guessed, the describer pressed any button on a wireless mouse held below the table, and a new word was displayed. At the beginning and the end of the experiment, as well as between each session, a calibration sequence was performed where the two interlocutors looked for 5 s at the left eye, right eye, nose, and mouth each, two times simultaneously following pre-recorded verbal prompts. This was done to prevent temporal drifts in gaze estimation accuracy. The auto-encoder network was retrained using data from the calibration sequences (*n* = 6·20·2). A final calibration sequence was not included in the optimization and was used for unbiased evaluation. The validation revealed a median error of 2.11° to the eye landmarks for the participants (experimenter: 1.42°). This error was lower error than in the previous implementation of the camera system, indicating the value of individual deep-learning calibration to actual face-to-face gaze. More detailed information about the eye-tracking technique can be found in Thorsson et al. ^42^. Missing data were defined as gaze that could not be estimated, and represented about 10.3% of the data collected in the experiment. Missing data could be caused by eye blinks, or by extensive head rotation of either the participant or the experimenter. Details about the eye-tracking accuracy, median error, precision, and movement stability can be found in Supplementary Information.

The game was played 4 rounds (90 s each), wherein the experimenter and participant presented (describer role) and listened twice. A maximum allowed time per word was set to 20 s to avoid too few trials.

After the experiment, to keep the experimenter blind to the results, we collected the full-scale AQ^54^ and the presence of self-reported eye contact discomfort^55^. The AQ is a self-administered questionnaire created to assess the extent to which an intellectually normal adult exhibits traits linked to the autism spectrum. Various research studies have explored the test–retest reliability and internal consistency of the AQ. For instance, Hoekstra et al. ^56^ reported satisfactory test–retest reliability and internal consistency in their study, with a test–retest correlation of 0.78 and Cronbach’s alphas of 0.81 and 0.71, in two separate samples. Another investigation by Broadbent et al. ^57^ reports Cronbach’s alphas of 0.84 for individuals with autism and 0.75 for individuals without diagnosis. These findings indicate that the AQ demonstrates robust test–retest reliability, signifying that the instrument yields a consistent measure of autistic traits. In our study, a four-point scale was used for the AQ scoring, following the implementations by Hoekstra et al.^56^.

### Data pre-processing

**Figure 2:**
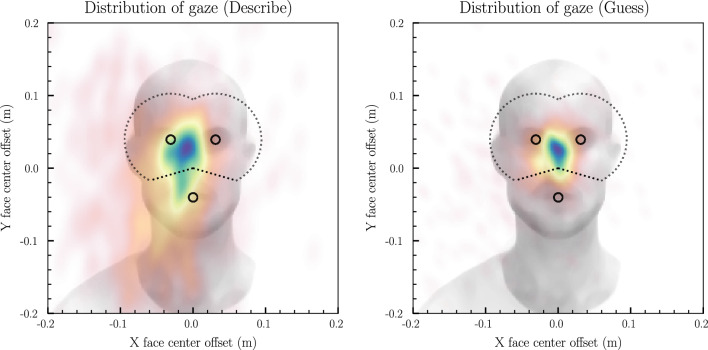
Kernel density estimations for the participants gaze coordinates in the two conditions. The dotted lines enclose the eye area of interest. Facial landmarks (left eye, right eye, and the mouth) used to define the area are shown as small black circles. *n* = 20.

A total of 4 trials in the condition where participants guessed and listened, and 3 trials in the condition where participants described words were removed when estimating the game features, due to misclicks. The last 60 s of one trial were excluded in estimating gaze percentages, due to technical error. The removal of these trials did not affect the main results. Gaze percentages were estimated as the average time spent looking at the eye area divided by the total time over the two trials when the participant described or guessed words. In order to estimate where the participants looked, facial planes (3D orientation and location) were first estimated based on facial landmark detection (see Thorsson et al. ^42^; Appendix A: 2.1 Estimation of face plane). Gaze vectors were then estimated based on a 3D gaze model, that included the head pose and the eye and pupil location. The gaze to the other person's face was estimated based on the average intersection of the two separate eye gaze vectors (from left and right eye) in the facial plane (see Supplementary Information and Thorsson et al. ^42^; Appendix A: 2.2 Line-plane intersection). The classification of eye gaze was based on the distance between the average intersection of the other individual's facial plane (meaning that the area moved with the participants/experimenter) and the eyes and mouth landmarks (see Thorsson et al. ^42^ and Supplementary Information). Following the previous implementation with the camera system^42^ we used a distance of 6.3 cm (~ 3°) to classify gaze to the eye area, see Fig. 2. We classified mutual eye contact as the instances when the data points of gaze, nearest in time, of participant and experimenter were both within the eye areas. The percentages of gaze to eye areas and mutual eye contact were then averaged first per trial, and then by condition.

### Statistical analysis

Multiple linear regression, ordinary least squares (OLS), were used to estimate the potential relationships between variables. Age and sex were included as covariates, in order to account for potential differences. Robust covariance matrices were used in all regressions to reduce any potential effects of heteroscedasticity, as motivated by Hayes and Cai ^58^.

### Ethics approval

The study was approved by the Swedish Ethical Review Authority (diary id: 2020-02076).

### Consent for participation

All participants and caregivers, if applicable, gave written consent for their child’s participation before the experiment. The individual who is identifiable in the images gave informed consent for publication.

## Results

### Eye gaze in the different communication roles

To examine eye gaze in face-to-face interaction, we looked at the percentages of gaze to the eye area and mutual eye contact across conditions.

The results indicated that when the participants listened/guessed words, they looked on average more than half of the time into the eyes of their interaction partner (mean ± SD: 55.8 ± 17.6%), which was significantly more than when they described words (37.1 ± 16.9%), *t*(19) = -4.62, *p* < 0.001.

**Figure 3:**
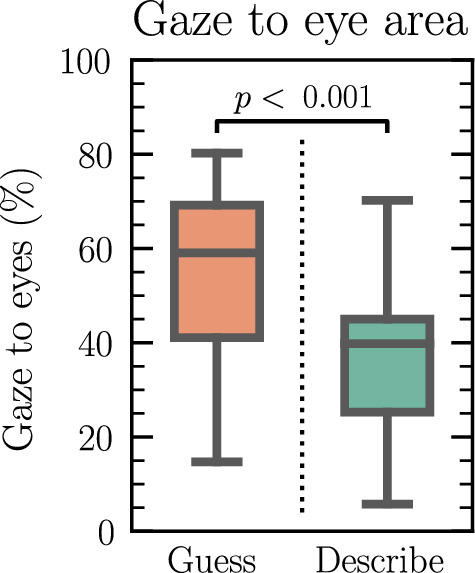
Boxplot to display the percentage of gaze to the experimenter’s eye area per condition. The box indicates the quartiles of the dataset, and the whiskers extend to represent the remainder of the distribution. *p*-value is from paired *t*-test. *n* = 20.

Of the registered gaze to the eye region, the proportion of eye gaze that was mutual eye contact between the participant and the experimenter was higher when they guessed words (27.2 ± 12.2%) than when they described words (21.2 ± 11.7%),* t*(19) = -2.67, *p* = 0.015. The gaze heatmaps are shown in Fig. 2*,* and boxplots for the gaze to the eye area per condition, are shown in Fig. 3*.* As a control analysis a paired *t*-test was performed to confirm that the difference in gaze between conditions was not driven by missing data, as for example caused by movement or extreme posture. There was no significant difference in missing data between conditions for the participants, *t*(19) = − 1.15, *p* = 0.26. More details about mutual eye contact and the role of the experimenter can be found in Supplementary Information.

### Influence of autistic traits and eye contact discomfort

**Figure 4:**
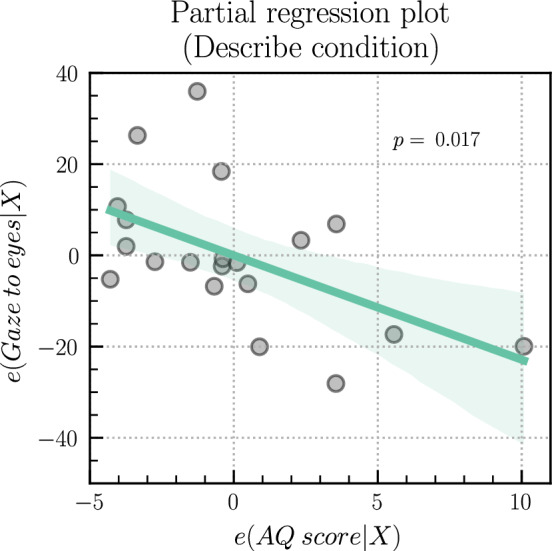
Partial regression plot, depicting the isolated impact of AQ scores on gaze to the eye while controlling for other factors. Points represent partial relationship and the green line shows the fitted regression model. The 95% CI is shown in faded green. *p*-value is from *t*-test.

In order to explore how individual differences in participant autistic traits and the presence or not of eye contact discomfort were associated with eye gaze data across conditions, we first conducted multiple linear regressions in the condition when the subjects described words. Sex was included as a variable in the regression analyses; however, it was not significant for any of the regressions, see Supplementary Information. This analysis revealed a significant negative linear relationship between AQ scores and the percentage of looking time at the experimenter's eye area (*β* = − 2.28, *t*(19) = − 2.67, *p* = 0.017, *η*^2^ = 0.25), meaning that for every 1-point increase in AQ, there was a ~ 2.3% decrease in gaze to the eyes of the experimenter (Fig. 4). The same pattern of results was observed for the proportion of gaze that was mutual (*β* = − 1.57, *t*(19) = − 2.71, *p* = 0.015, *η*^2^ = 0.30).

In regards to the effect of self-reported eye contact discomfort, results followed the same pattern, but here eye contact discomfort was not significantly related to eye gaze (*β* = − 12.95, *t*(19) = − 1.47, *p* = 0.16, *η*^2^ = 0.15) nor mutual eye contact (*β* = − 10.07, *t*(19) = − 1.83, *p* = 0.056, *η*^2^ = 0.10).

Next, we examined the associations between AQ and eye contact discomfort in the condition where the subjects were listening/guessing using the same covariates. In contrast to what was observed when the subjects described words, in the listening/guessing condition we found no significant relationships between AQ scores and the percentage of gaze to the eye area of the experimenter (*β* = − 1.91,* t*(19) = − 9.78, *p* = 0.34, *η*^2^ = 0.06) nor mutual eye contact (*β* = − 0.94, *t*(19) = − 0.71, *p* = 0.49, *η*^2^ = 0.03). This means that when listening/guessing what the experimenter was trying to communicate, neither the participants’ percentage of gaze to the eye area of the experimenter nor mutual eye contact was associated with autistic traits.

Eye contact discomfort was reported by six of the twenty participants. When comparing this subgroup with the other participants who did not report issues with eye contact, in the condition where participants listened/guessed words, we found a significant reduction of gaze to the eyes of the experimenter (*β* = − 22.83, *t*(19) = − 2.43, *p* = 0.027, *η*^2^ = 0.27) and mutual eye contact (*β* = − 15.11, *t*(19) = − 2.13, *p* = 0.049, *η*^2^ = 0.22) in those who were reporting eye contact discomfort. This means that, on average, those who reported eye contact discomfort gazed 22.8% less into the experimenter's eyes and shared 15.1% less mutual eye contact, when accounting for age and sex.

To summarize, we found a negative association between AQ scores and the percentage of eye gaze and mutual eye contact when participants were describing words, but not when they listened and guessed words. Further, we found that in the listening/guessing condition, the sub-group of participants who reported eye contact discomfort demonstrated a significant reduction in the amount of gaze to the experimenter’s eye area as well as a reduction in mutual eye contact.

## Discussion

In this study, we aimed to examine how eye gaze and mutual eye contact manifest during face-to-face communication, as well as how this behavior relates to autistic traits and communication role. This was made possible with the use of a novel minimally intrusive eye-tracking device with deep-learning methodology^42^ while participants and the experimenter were engaged in a referential communication task.

First, results showed that the participant’s role during the referential communication task modulated the eye gaze and eye contact behavior in different ways. Specifically, the participants gazed less to the eye area of the experimenter when they were describing words compared to when they were guessing/listening. Additionally, we found that individuals with high autistic traits demonstrated reduced eye gaze and mutual eye contact, particularly when describing words for the experimenter.

Moreover, the current study also presents one of the first efforts to examine self-reported discomfort with eye contact during face-to-face interactions (see also Tönsing et al.^19^ who looked at self-reported gaze anxiety). Results in this regard show that the participants who reported eye contact discomfort had less eye gaze and mutual eye contact when listening/guessing what their partner was communicating. This implies that for some participants, self-perceived discomfort with eye contact may drive the reduction across some, but perhaps not all communicative situations. More extensive research is needed to further examine the specificity of this type of discomfort-driven behavior.

Finally, results showed that eye contact was a relatively common behavior during the interaction. Specifically, we found that participants engaged in mutual eye contact 27.2% of the interaction in the condition when the participants guessed words and 21.2% when they described referents to the experimenter. These percentages are higher than the 3.5% found in a study examining face-to-face interaction using eye-tracking glasses^33^, and 12.5% in a study that used commercial table-based eye trackers and cameras placed behind participants^19^. The gaze behavior of the experimenter was largely similar to that of the participant (see Supplementary Information). Several factors could have played a role in the variability in percentages of eye gaze and eye contact behavior across studies. Differences may stem from differences in experimental setups. The eye-tracking setup used in our study was developed with neurodevelopmental symptoms in mind and avoided using (potentially distracting) wearables; it did not either use visible devices behind or above the participants^37,59,60^. Because we only used small cameras (*ø* = 8 mm) placed on the table between the participants, without any other cameras or eye trackers in the room, we reduced distractions from nearby objects, which may encourage participants to concentrate on the task and their conversation partners.

Another aspect to consider is the difference in AOI sizes. Our AOI sizes were observably larger than those implemented by Ross et al. ^17^ and Tönsing et al. ^19^ but smaller than those used by Holleman et al. ^61^, for example. Our selection of AOIs was based on our previous implementation of the camera system^42^, with considerations made for estimated accuracy and the anticipation of data noise. We aimed to maximize AOI sizes without excessively extending beyond the eye area, thereby minimizing the risk of false positives^62^. However, it is crucial to note that smaller AOIs generally reduce false classifications, provided accuracy remains adequate. Conversely, larger AOIs may heighten the risk of false positives, which could contribute to the relatively high amount of observed eye gaze in our experiment.

Yet another factor of consideration is the definition of eye contact. We used the proportion of the total number of data points within the eye area as a proportion of total time, similar to other mentioned implementations in face-to-face interactions^19,25^. The study with the lowest described gaze percentage, however, seemed to only include data categorized as fixations^33^.

Furthermore, factors such as data quality could also affect the result. Tönsing et al. ^19^, report low (1.3%) missing data during their validation sequences, similar to ours, but they present no information on missing data during the full experiment. Mayrand et al. ^33^ also did not report this. Nonetheless, the exclusion of individuals based on data quality can influence the results. Notably, no participants were excluded on this basis from our analysis.

An important aspect of our deep-learning-based gaze estimation is that it provides 3D gaze vectors in world coordinates, intersecting the facial plane of the other person estimated in world coordinates^42^. An error known as the parallax is generally present for commercial table-based eye trackers^25,26,41^, which are calibrated to estimate 2D gaze coordinates located on a specific plane, often limited to a specific distance. This error is further exaggerated when including another scene or wearable camera as with eye-tracking glasses^63^, adding another parallax error when capturing another perspective than the calibrated plane. Our gaze estimation relies on 3D gaze vectors' average intersection point with the participant's facial plane, which itself is estimated in three dimensions. With this approach, unlike glint-based eye trackers, we do not encounter parallax issues normally due to estimations in a fixed plane. While we did not systematically investigate the stability of gaze estimation during all types of head movement throughout the specific experimental conditions, we minimized discrepancies by consistently collecting data for neural network training throughout the experiment, using brief calibrations between changing conditions. Given these methodological precautions, differences in the gaze behavior between conditions are unlikely to solely reflect movement differences, as for example, extreme movement was eliminated in data loss and there were no differences in data loss between conditions (see Supplementary Information). Another possibility for the difference in the amount of mutual gaze with previous research may be due to the nature of the referential task itself. The task in the present study required a mutual and consecutive exchange of information to successfully achieve the goal (correct guess) and involved both the production and interpretation of verbal and nonverbal communication, such as eye contact. It designated each participant to take a specific, and clearly defined, role in the communicative context with both the participant and the experimenter consecutively engaging in both roles. Because the participant intended to describe a word that was unknown to the experimenter in order for him to correctly guess what was being communicated our task differed in comparison to other recent face-to-face gaze studies^17,19^ where participants were asked to provide a definition to a word on a paper shown by the experimenter^64^ or with the experimenter listening or answering questions in a semi-structured interview^19^.

In relation to the task, the results of the present study suggest that the participant role in the verbal task influences eye gaze and eye contact behavior of the participants with the experimenter. We find that participants gazed more at the experimenter's eyes when they were listening/guessing than when they were describing the referent, suggesting more social attention and joint engagement when encoding verbal information than when providing verbal input^65^. These findings align with well-replicated findings from research on nonverbal face-to-face communication: listeners tend to maintain longer gaze at speakers, while speakers gaze back less^4–7^. These findings also support the hypothesis introduced in the initial discussion on cognitive load, which has been associated with decreased eye gaze as a strategy to strategy to lower the load^9,66,67^.

## Limitations and strengths

Some limitations and strengths of the current study are notable. In terms of limitations, first, it is important to point out that we had a relatively limited sample size (*n* = 20), which highlights the need for more research conducted in larger samples. That said, our sample size was comparable with other studies using a similar design^17,25,68^, and a smaller sample size may be sufficient, and more feasible when analyzing this type of highly data-intensive material^17^. Moreover, our experiment was of course also not a typical face-to-face interaction, as occurs in daily life, since the participants were aware that they were taking part in an experiment, and the task was based on a classic word game. Furthermore, one of the interlocutors, the experimenter, was the same across participants. While this prevented us from exploring interactions between individuals of different levels of autistic traits^16^, it may have provided more comparable settings for participants compared to participant-only dyads. Overall, in terms of eye gaze and movement, the experimenter’s behavior did not seem to diverge from the participants’ behavior (see Supplementary Information). Nevertheless, other factors, such as the experimenter's repeated performance with the task, may influence his behavior, compared to participant. Future research could consider recruiting dyads, which would assist in controlling this potential influence but would also include other aspects of variability. In terms of strengths, as previously discussed, the camera system used in our study^42^ was developed using a deep-learning methodology which is relatively robust to movement^42,49^. It was also set up to explore patterns of mutual eye gaze in people with varying levels of neurodevelopmental traits, meaning that it was intended to be minimally distracting, and did not include any wearable components or additional cameras in the room^3,69^. The use of our adapted camera system, together with the ability to capture two-sided mutual eye gaze, may have added to the validity of our findings and allowed us to be more inclusive of individuals with higher autistic traits. Relatedly, using the dimensional analysis of AQ scores allowed us to capture more nuances of autistic traits in the population^47,56,70^. Lastly, having the participant fill out questionnaires after the interaction task reduced the possibility of priming while having the experimenter be blind to the AQ scores and eye contact discomfort reduced potential bias.

## Conclusions

Taken together, in comparison to prior studies utilizing different eye-tracking technologies, experimental setups and tasks, our findings reveal not only important similarities but also add some intriguing novelties. Adding to the current state of research in this area, we show that eye gaze data measured with non-wearable face-to-face eye tracking and supported by deep learning computations is related to individual differences in autistic traits (AQ) and to eye contact discomfort, and dependent on the individual’s communicative role.

### Supplementary Information

Supplementary Information.

## Data Availability

The study reported in this article was not pre-registered. The data have not been made available on a permanent third-party archive because participants were not asked to consent for their data to be made publicly available, even anonymized. Data are available upon request from those who wish to collaborate with us, via a visitor agreement with the University of Gothenburg, if appropriate, under existing ethics approval.
