# Supplementary material for: Influence of autistic traits and communication role on eye contact behavior during face-to-face interaction
Source: Sci Rep. 2024 Apr 8;14:8162. doi: 10.1038/s41598-024-58701-8 (PMC11001951; doi:10.1038/s41598-024-58701-8)
Supplement: Supplementary file 1 — Supplementary Information. [file 41598_2024_58701_MOESM1_ESM.pdf]

Supplementary Information

Influence of autistic traits and communication role on eye contact behavior during face-to-face interaction

Max Thorsson[1✉], Martyna A. Galazka[1,2], Jakob Åsberg Johnels[1,3], and Nouchine Hadjikhani[1,4]

[1] Gillberg Neuropsychiatry Centre, Institute of Neuroscience and Physiology, University of Gothenburg, Gothenburg, Sweden
[2] Division of Cognition and Communication, Department of Applied Information Technology, University of Gothenburg, Gothenburg, Sweden
[3] Section of Speech and Language Pathology, Institute of Neuroscience and Physiology, University of Gothenburg, Gothenburg, Sweden
[4] Athinoula A. Martinos Center for Biomedical Imaging, Massachusetts General Hospital, Harvard Medical School, Boston, MA, USA

✉ Corresponding author (max.thorsson@gu.se)

Supplementary Content

The supplementary text serves as an extended method to the main paper comprising information about eye tracking quality, effects of movement, experimenter role, mutual eye contact, and supplementary tables.

1. Eye-tracking quality

Accuracy, median error, precision, and data loss were estimated over the gaze to the eye area, during calibration sequences that were not included in neural network optimization, and during the episodes of role switching. Accuracy is quantified as the average deviation in degrees of visual angle from the landmark-based location on the facial plane of the individual being gazed at. Median error, on the other hand, represents the rank-based middle value of the deviations in degrees of visual angle from the same landmark-based location. Precision is defined as the standard deviation (*SD*) in degrees of visual angle degrees, based on the estimated gaze coordinates intersecting with the facial plane. Missing data are defined as gaze that could not be estimated such as during eye blinks, or to extensive head rotation of either participant or experimenter. The training metrics were computed during calibration, over twelve 5-second intervals (6 · left and right eye) of gazing at each eye (left and right). The unbiased evaluation metrics were computed over everyone's two final 5-second intervals of gazing at each eye (2 · left and right eye). Data was averaged over eyes and per individual's trials per metric.

On average, less than 1% (0.077% and 0.019%) of both interlocutors' gaze data were missing during the training data sequences included in the neural network optimization. Similarly, less than 1% (0.043% and 0.000%) of data were missing during the validation of gaze to the eyes. Details on accuracy and precision during the unbiased evaluation can be found in *Supplementary Table S1*, and the training data between changes of roles in the experiment can be found in *Supplementary Table S2*.

Supplementary Table S1 Eye tracking median error, accuracy, and precision in degrees of visual angle in the *unbiased evaluation*, $n_{particpant\ trials} = 20$, $n_{experimenter\ trials} = 20$.

	**Median error**		**Accuracy**		**Precision**	
	Participants	*Experimenter*	*Participants*	*Experimenter*	*Participants*	*Experimenter*
Median	2.11	1.42	2.41	1.98	1.97	1.27
Mean	2.06	1.65	2.56	2.27	2.05	2.16
SD	0.54	0.74	0.70	0.93	1.15	1.73

Supplementary Table S2 Eye tracking median error, accuracy, and precision in degrees of visual angle in *training data* between changes of roles in the experiment, $n_{particpant\ trials} = 20$, $n_{experimenter\ trials} = 20$.

	**Median error**		**Accuracy**		**Precision**	
	Participants	*Experimenter*	*Participants*	*Experimenter*	*Participants*	*Experimenter*
Median	1.30	1.54	1.86	1.96	2.31	1.78
Mean	1.39	1.54	2.08	2.03	2.40	1.85
SD	0.41	0.27	0.61	0.31	1.05	0.40

2. Effects of movement

Following with the approach outlined by Vehlen, et al. [1] (but adapted for our particular data and design), we operationalized movement as the standard deviation (*SD*) for the estimated 3D positions (*x*, *y*, and *z*) of facial landmarks, measured in degrees of visual angle. This formulation can be articulated mathematically:

$$Movement\ index = \frac{180}{\pi} \cdot arctan\left(\frac{\left(\frac{SD(x)+SD(y)+SD(z)}{3}\right)}{mean\ distance\ between\ individuals}\right)$$

Here, the movement index was computed over each individual's two 5-second intervals of gazing at each eye (left and right) during calibration. The validation data used were distinct from those employed in the calibration, i.e., the neural network training. This method allowed us to quantify movement based on facial landmark variability while maintaining independence from the training dataset.

In order to test if head movement of the other person was affecting data quality, we examined if there were any significant correlations between head movement, of both the person gazed at and participant, and eye tracking accuracy during the gaze to the eye areas. Spearman's rank correlation coefficient was used due to the presence of extreme values in the movement data. Upon analysis, we found that neither the participants' nor the experimenter's movement yielded statistically significant effects on correlations with the participants' accuracy (experimenter movement: $r(19) = 0.28, p = 0.24$, participant movement: $r(19) = 0.41, p = 0.07$). This suggests that variations in movement did not notably impact the accuracy of the validation results to a considerable degree. Movement indices were on average 0.13° for participants and 0.15° for the experimenter during the sequences for validation of eye gaze.

We also performed analyses on differences in movement in the two conditions. Our analysis revealed differences in the amount of movement between experimental conditions. Specifically, participants demonstrated significantly greater movement when describing words compared to when guessing them, $t(19) = 4.82, p < 0.001$. On average, participants had a mean movement index of 1.08° (± 0.38°) when describing the word and 0.69° (± 0.30°) when guessing.

To explore whether the autism spectrum quotient (AQ) scores were co-varying with motion, we conducted a control correlation analysis between AQ and the movement indices. As depicted in *Supplementary Table S3*, the results indicate no significant relationship between participant (or experimenter, *p*s ≥ 0.22) movement with their AQ scores (*p*s ≥ 0.74). This confirms that the amount of movement was not significantly related to the AQ scores, which strengthens the validity of our conclusions.

Supplementary Table S3 Spearman's rank correlation coefficients between AQ and the amount of movement, $n_{particpant\ trials} = 20$, $n_{experimenter\ trials} = 20$.

	**Participant describing**	**Participant guessing**
Participant movement	$r = 0.08, p = 0.74$	$r = 0.06, p = 0.80$
Experimenter movement	$r = 0.29, p = 0.22$	$r = 0.13, p = 0.58$

Finally, we estimated whether the amount of missing data in the gaze to the eye area, due to movement artifacts in the participants, differed between conditions. This was done to ensure that any excessive movement, which could result in missing data, was not the underlying cause of the disparity in gaze between the conditions. We found no significant differences in the missing data between conditions, $t(19) = 1.15, p = 0.26$ (Describe: 8.5 ± 4.4%; Guess: 11.0 ± 11.2%). Note, that the proportion of missing data for the participants also included when the experimenter's facial plane couldn't be detected.

3. Experimenter role and mutual eye contact

Experimenter gaze

We conducted several sub-analyses in order to provide more information about the experimenter's role in the experiment. In terms of the experimenter's role and behavior, we explored whether his behavior and recording diverged from that of the participants. In general, this was not the case. Specifically, his average amount of missing data, when he described words was higher (missing data: + 4.9%) compared to when participants guessed words but within ± 1.2 *SD* of the participants' values; his amount of eye gaze in the guessing condition was 43.4%; his amount of eye gaze in the describing condition was 55.8%.

Mutual eye contact

As a control analysis, we tested if the mutual eye contact between the participants and the experimenter was higher than random in the two conditions. The *estimated* proportion of mutual eye contact, caused by random gaze patterns, would theoretically be the product, i.e., the intersection, of the two probabilities (here the percentages) of eye contact, expressed by, $P(A_{\text{gaze\_eyes}}) \cdot P(B_{\text{gaze\_eyes}}) = P(A \cap B_{\text{eye\_contact}})$. Related t-tests revealed that the proportion of mutual eye contact was significantly higher than random in both the guessing, $t(19) = 4.70$, $p < 0.001$, and the describing conditions, $t(19) = 5.84$, $p < 0.001$. This means that the mutual eye contact was not a result of random gaze patterns from each individual, but a specific process driven by mutual timed gaze to the eye areas of the dyad.

Order effect

A control analysis in order to rule out any potential effects of repeated performance of the word task of the experimenter would impact our results related to AQ. The analysis showed no significant association between participant order number and AQ scores, $r(19) = 0.30$, $p = 0.20$. This suggests that any potential effect from the experimenter's repeated involvement is unlikely to influence our results related to autistic traits.

4. Supplementary Tables

Tables include the dependent (Dep.) variable, the ordinary least squares (OLS) models total and adjusted (Adj.) R^2, F-statistic, model significance, covariance type, number of (No.) observations, degrees of freedom (Df), intercept, predictors, standard error (std err), and t-statistic (t), measuring the significance of coefficient (coef). The p-value from a t-test (p>|t|), and the boundary values of the 95% confidence interval ([0.025, 0.975]) for the coef. In the following tables, the autism spectrum quotient score is denoted as "AQ" and self-reported eye contact discomfort as "ECD". Note that heteroscedasticity robust covariance estimators (HC3) were used to calculate standard errors.

Describing condition

Supplementary Table S4 Regression results for eye gaze during describing condition, AQ and eye gaze.

**Dep. Variable:**	eye gaze	**R-squared:**	0.401
**Model:**	OLS	**Adj. R-squared:**	0.289
**No. Observations:**	20	**F-statistic:**	4.938
**Df Residuals:**	16	**Prob (F-statistic):**	0.0129
**Df Model:**	3	**Covariance Type:**	HC3

	**coef**	**std err**	**t**	**p>|t|**	**[0.025**	**0.975]**
**Intercept**	75.0605	37.294	2.013	0.061	-4.000	154.121
**age**	-0.0035	0.878	-0.004	0.997	-1.865	1.858
**AQ**	-2.2869	0.857	-2.667	0.017	-4.105	-0.469
**sex**	16.8278	7.414	2.270	0.037	1.110	32.546

Supplementary Table S5 Regression results for eye gaze during describing condition, AQ and eye contact.

**Dep. Variable:**	eye contact	**R-squared:**	0.259
**Model:**	OLS	**Adj. R-squared:**	0.120
**No. Observations:**	20	**F-statistic:**	2.933
**Df Residuals:**	16	**Prob (F-statistic):**	0.0653
**Df Model:**	3	**Covariance Type:**	HC3

	**coef**	**std err**	**t**	**p>|t|**	**[0.025**	**0.975]**
**Intercept**	51.0049	24.178	2.110	0.051	-0.250	102.260
**age**	-0.0463	0.609	-0.076	0.940	-1.338	1.246
**AQ**	-1.5711	0.579	-2.713	0.015	-2.799	-0.344
**sex**	5.4089	5.351	1.011	0.327	-5.935	16.753

Supplementary Table S6 Regression results for eye gaze during describing condition, ECD and eye gaze.

**Dep. Variable:**	eye gaze	**R-squared:**	0.285
**Model:**	OLS	**Adj. R-squared:**	0.150
**No. Observations:**	20	**F-statistic:**	2.381
**Df Residuals:**	16	**Prob (F-statistic):**	0.108
**Df Model:**	3	**Covariance Type:**	HC3

	**coef**	**std err**	**t**	**P>|t|**	**[0.025**	**0.975]**
**Intercept**	32.8945	24.271	1.355	0.194	-18.558	84.346
**age**	0.1193	0.845	0.141	0.889	-1.672	1.911
**ECD**	-12.9489	8.787	-1.474	0.160	-31.577	5.679
**sex**	11.8682	7.505	1.581	0.133	-4.041	27.778

Supplementary Table S7 Regression results for eye gaze during describing condition, ECD and eye contact.

**Dep. Variable:**	eye contact	**R-squared:**	0.178
**Model:**	OLS	**Adj. R-squared:**	0.024
**No. Observations:**	20	**F-statistic:**	1.343
**Df Residuals:**	16	**Prob (F-statistic):**	0.296
**Df Model:**	3	**Covariance Type:**	HC3

	**coef**	**std err**	**t**	**p>|t|**	**[0.025**	**0.975]**
**Intercept**	22.8683	16.278	1.405	0.179	-11.640	57.376
**age**	0.0226	0.579	0.039	0.969	-1.205	1.250
**ECD**	-10.0740	5.513	-1.827	0.086	-21.761	1.613
**sex**	1.9031	5.393	0.353	0.729	-9.530	13.337

Listening/guessing condition

Supplementary Table S8 Regression results for eye gaze during listening/guessing condition, AQ and eye gaze.

**Dep. Variable:**	eye gaze	**R-squared:**	0.194
**Model:**	OLS	**Adj. R-squared:**	0.043
**No. Observations:**	20	**F-statistic:**	0.5969
**Df Residuals:**	16	**Prob (F-statistic):**	0.626
**Df Model:**	3	**Covariance Type:**	HC3

	**coef**	**std err**	**t**	**p>|t|**	**[0.025**	**0.975]**
**Intercept**	84.1053	47.127	1.785	0.093	-15.800	184.011
**age**	0.2685	0.807	0.333	0.744	-1.442	1.979
**AQ**	-1.9100	1.954	-0.978	0.343	-6.051	2.231
**sex**	3.2683	10.660	0.307	0.763	-19.330	25.866

Supplementary Table S9 Regression results for eye gaze during listening/guessing condition, AQ and eye contact.

**Dep. Variable:**	eye contact	**R-squared:**	0.085
**Model:**	OLS	**Adj. R-squared:**	-0.087
**No. Observations:**	20	**F-statistic:**	0.2484
**Df Residuals:**	16	**Prob (F-statistic):**	0.861
**Df Model:**	3	**Covariance Type:**	HC3

	**coef**	**std err**	**t**	**p>|t|**	**[0.025**	**0.975]**
**Intercept**	46.1283	33.098	1.394	0.182	-24.037	116.293
**age**	-0.0240	0.625	-0.038	0.970	-1.348	1.300
**AQ**	-0.9355	1.323	-0.707	0.490	-3.740	1.869
**sex**	-0.0261	7.684	-0.003	0.997	-16.315	16.263

Supplementary Table S10 Regression results for eye gaze during listening/guessing condition, ECD and eye gaze.

**Dep. Variable:**	eye gaze	**R-squared:**	0.393
**Model:**	OLS	**Adj. R-squared:**	0.280
**No. Observations:**	20	**F-statistic:**	2.532
**Df Residuals:**	16	**Prob (F-statistic):**	0.0938
**Df Model:**	3	**Covariance Type:**	HC3

	**coef**	**std err**	**t**	**p>|t|**	**[0.025**	**0.975]**
**Intercept**	57.3756	25.616	2.240	0.040	3.072	111.679
**age**	0.2130	0.775	0.275	0.787	-1.430	1.856
**ECD**	-22.8332	9.398	-2.430	0.027	-42.756	-2.910
**sex**	-1.8787	7.889	-0.238	0.815	-18.602	14.845

Supplementary Table S11 Regression results for eye gaze during listening/guessing condition, ECD and eye contact.

**Dep. Variable:**	eye contact	**R-squared:**	0.330
**Model:**	OLS	**Adj. R-squared:**	0.205
**No. Observations:**	20	**F-statistic:**	1.771
**Df Residuals:**	16	**Prob (F-statistic):**	0.193
**Df Model:**	3	**Covariance Type:**	HC3

	**coef**	**std err**	**t**	**p>|t|**	**[0.025**	**0.975]**
**Intercept**	35.8125	22.132	1.618	0.125	-11.106	82.731
**age**	-0.1028	0.664	-0.155	0.879	-1.510	1.304
**ECD**	-15.1141	7.082	-2.134	0.049	-30.127	-0.101
**sex**	-2.8756	6.351	-0.453	0.657	-16.339	10.588

5. Supplementary Figures

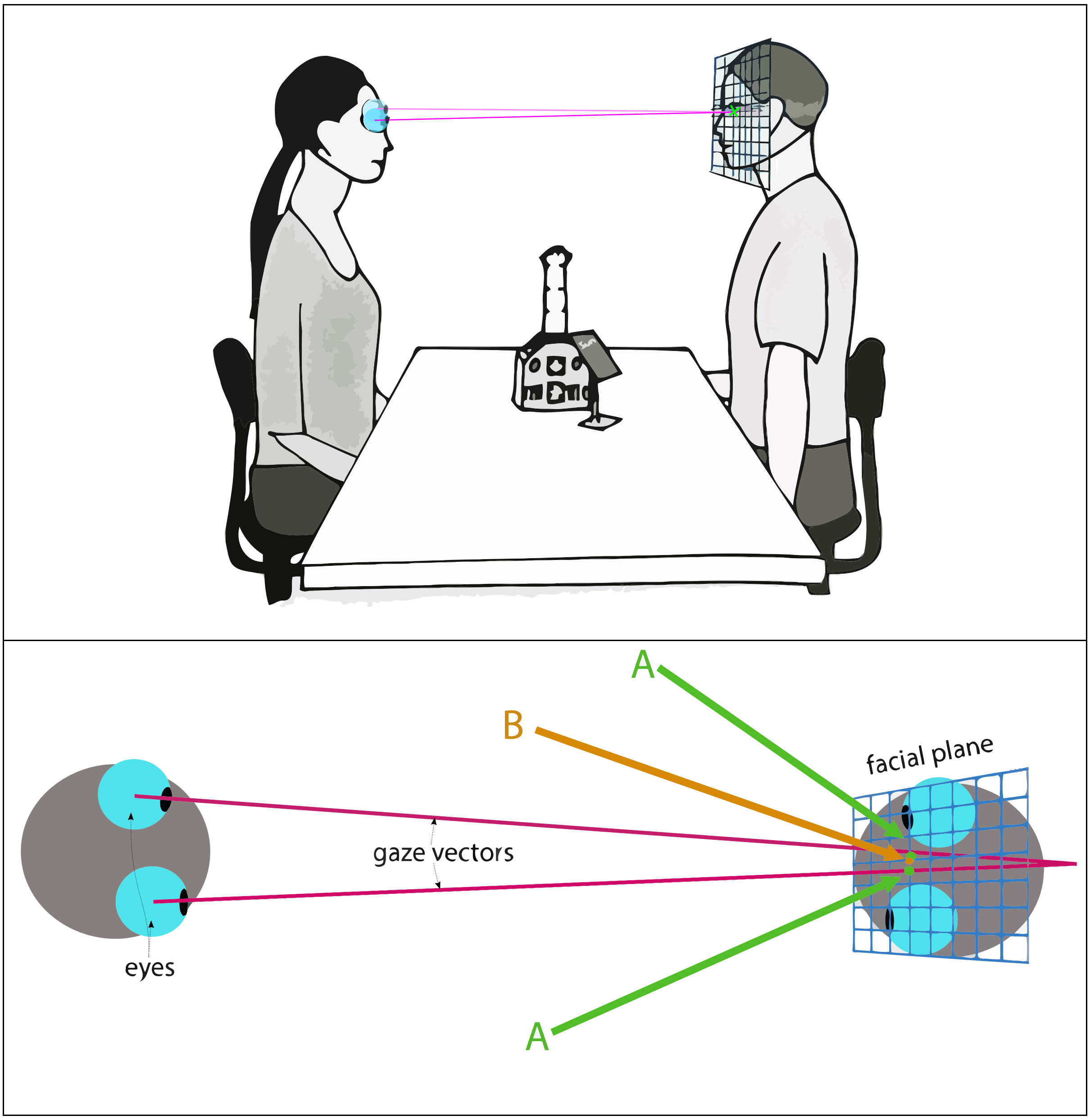

Supplementary Figure S1 On the top panel, the figure shows the gaze vectors (pink lines) intersecting the facial plane of the opposing person. On the lower panel, you can see the details of how the vectors (green lines, A) result in the average point of intersection (yellow line, B) which is the data point used to calculate gaze.
